# Selective Consumption of Fish Oil at End of the Day Increases the Physiological Fatty Acid Compositions of Eicosapentaenoic Acid and Docosahexaenoic Acid in Mice

**DOI:** 10.3390/molecules27041271

**Published:** 2022-02-14

**Authors:** Hiroki Matsuzaka, Hiroki Matsuyama, Wataru Tanaka, Hayato Tajiri, Hiroyuki Sakakibara

**Affiliations:** 1 Faculty of Agriculture, University of Miyazaki, 1-1 Gakuen-kibanadai Nishi, Miyazaki 889-2192, Japan; m1hi2ro2ki5@theia.ocn.ne.jp; 2 Graduate School of Agriculture, University of Miyazaki, 1-1 Gakuen-kibanadai Nishi, Miyazaki 889-2192, Japan; nb21008@student.miyazaki-u.ac.jp (H.M.); gc14026@student.miyazaki-u.ac.jp (W.T.); gc16021@student.miyazaki-u.ac.jp (H.T.)

**Keywords:** high-fat diet, mice, overweight, omega-3/omega-6 ratio, selectivity

## Abstract

Diets with high daily fat consumption are associated with excess weight. However, the effects of fat type and consumption timing on excess weight remain unclear. We investigated the selection of a 30% (*w*/*w*) fat diet of soybean oil (SOY), lard (LARD), and fish oil (FISH) on the metabolic parameters of mice. Male C57BL/6 mice were divided into the double SOY-box (w-SOY), SOY-box/LARD-box (SOY-vs-LARD), or SOY-box/FISH-box (SOY-vs-FISH) groups and allowed to selectively consume for 8 weeks. The total energy intake was similar for all groups, but the mice selectively chose to consume LARD over SOY and SOY over FISH. Body weight in the SOY-vs-LARD group was significantly higher than that in the w-SOY and SOY-vs-FISH groups. Additionally, minimal but selective consumption of an omega-3 fatty-acid-rich FISH diet at the end of the active period increased the physiological fatty acid compositions of eicosapentaenoic acid (EPA) and docosahexaenoic acid (DHA) in the SOY-vs-FISH group; their metabolic parameters were also lower than the SOY-vs-LARD group. In conclusion, selectively consuming small amounts of fish oil at the end of the day may prevent excess weight compared with LARD consumption.

## 1. Introduction

Life insurance data indicate that obesity is associated with increased mortality owing to highly elevated risks of adverse health outcomes such as type 2 diabetes mellitus [1,2]. The combined medical costs associated with treating obesity-related diseases are expected to increase by USD 48–66 billion annually in the US and GBP 1.9–2.0 billion annually in the UK by 2030 [3]. Therefore, implementing effective policies to promote healthier weights may yield economic benefits.

Nutrition transition can be defined as shifts in food habits, and is characterized by high-fat (chiefly saturated animal fat) and hypercaloric food consumption [4]. High daily fat consumption is associated with being overweight or obese, thus leading to increased mortality [5]. Increased dietary fats have been associated with elevated energy intake and adiposity [6]. In addition to total fat consumption, individual fatty acid compositions are considered when studying dietary fats and obesity [7]. For example, mice who consumed a high-fat diet containing Spirulina lipids (46.8% palmitic acid, 20.3% γ-linolenic acid, and 16.8% linoleic acid) or fish oil (26.6% docosahexaenoic acid [DHA], 14.3% eicosapentaenoic acid [EPA], and 14.4% palmitic acid), gained less weight than mice consuming the same amount of a high-fat diet composed of soybean oil (54.5% linoleic acid and 21.2% oleic acid) or lard (45.1% oleic acid and 26.5% palmitic acid) without affecting their daily food intake [8]. Additionally, body weights of mice that consumed a high fat diet containing microalgal oil (56.8% DHA, 22.4% palmitic acid) were significantly decreased compared with the mice that consumed fish oil [9]. Du et al. conducted a meta-analysis of randomized controlled trials and found that fish oil supplementation may help reduce abdominal fat [10]. These findings suggest that some fats may help prevent excess weight gain and obesity.

Animals, including rodents and humans, tend to preferentially select fatty foods for their good flavor and texture [11]. Takeda et al. reported that in a two-bottle choice test, mice preferred vegetable oils [12]. When comparing *ad libitum* food intake using the same percentages of fats from different sources, mice consumed similar amounts of soybean-oil-based and lard-based diets, but markedly less of the fish-oil-based diet [13].

Here, we had doubts as to whether these preferences observed in the presence of plural fat-based diets indicate diurnal differences and affect weight gain. The results may provide insight into preventing excess weight gain and obesity from the viewpoint of fat origins, including fatty acid composition. Thus, we investigated the effects of selecting high-fat diets composed of soybean oil, lard, and fish oil on metabolic parameters in mice using a self-selection regimen from two diet boxes.

## 2. Results

### 2.1. Preferential Consumption of High-Fat Diets Composed of Different Fat Sources

**Figure 1:**
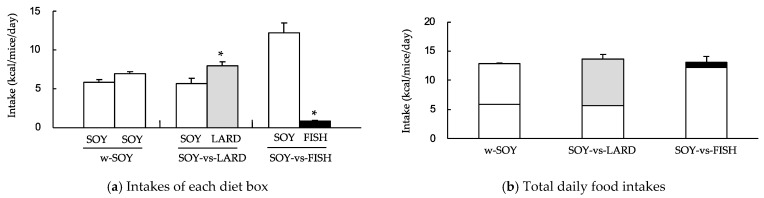
Selectivity for high-fat diets composed of different fat sources in the two-diet-box choice test. Mice were given two diet boxes per cage: either two 30% (*w*/*w*) soybean-oil-based high-fat (SOY) diet boxes (w-SOY); a SOY-diet box and a 30% (*w*/*w*) lard-based high-fat (LARD) diet box (SOY-vs-LARD); or a SOY-diet box and a 30% (*w*/*w*) fish oil-based high-fat (FISH) diet box (SOY-vs-FISH). The mice were permitted free access to deionized water and both diet boxes. (**a**) Intake of each diet box was measured. (**b**) Intake from both diet boxes was summed to yield the total daily food intake. Data are shown as the mean ± SD (*n* = 3). * *p* < 0.01 vs. SOY diet box.

Mice in the SOY-vs-LARD group consumed significantly more of the LARD diet (8.0 ± 0.5 kcal/mice/day) than the SOY diet (5.7 ± 0.7 kcal/mice/day), and mice in the SOY-vs-FISH group consumed significantly more of the SOY diet (12.3 ± 1.3 kcal/mice/day) than the FISH diet (0.8 ± 0.1 kcal/mice/day) (Figure 1a). No remarkable difference was observed in the w-SOY group. The total daily energy intake (the sum of both diet boxes) was similar for all three groups (Figure 1b). Daily consumptions of individual fatty acids are summarized in Table 1. The SOY-vs-LARD group consumed significantly higher quantities of saturated fatty acids and monounsaturated fatty acids, and lower quantities of omega-6 fatty acids than those of w-SOY and SOY-vs-FISH groups. Daily consumption of omega-3 fatty acids in the SOY-vs-FISH group was significantly higher than that of the SOY-vs-LARD group, and remarkably higher than the w-SOY group. Omega-6/omega-3 consumption ratios of w-SOY, SOY-vs-LARD, and SOY-vs-FISH groups were 10.2, 13.0, and 7.3, respectively (Table 1).

### 2.2. Diurnal Feeding Patterns

**Figure 2:**
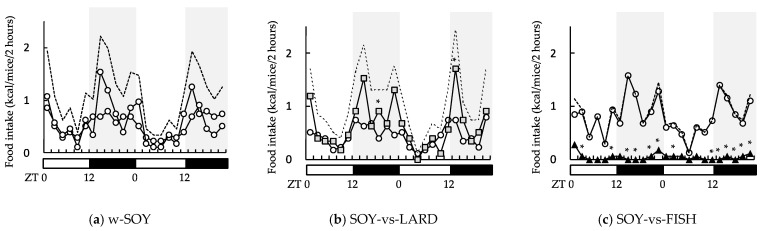
Effects of selectivity for high-fat diets composed of different fat sources on diurnal food intake rhythms. Mice were given two diet boxes: (**a**) w-SOY; (**b**) SOY-vs-LARD; and (**c**) SOY-vs-FISH. Food intake was averaged every 2 h (*n* = 3) for 48 h. ○, SOY; ■, LARD; ▲, FISH; dashed line, sum intake. * *p* < 0.01 vs. SOY diet group.

During week 7, the food intake was quantified at 2 h intervals. Figure 2 illustrates the evolution of the food intake, expressed in energy (kcal) per mouse, during the 48 h period. The total food intake (dashed line) shows similar oscillation patterns for the w-SOY (a), SOY-vs-LARD (b), and SOY-vs-FISH (c) groups. Comparing the two diet boxes revealed remarkable differences in the SOY-vs-LARD and SOY-vs-FISH groups. First, mice preferentially consumed the LARD diet, with a clear oscillation pattern, in the SOY-vs-LARD group. Second, the oscillation pattern in the SOY-vs-FISH group resulted from selective consumption of the SOY diet. Mice consumed the FISH diet at the end of the dark period (active phase), although the amount was minimal.

### 2.3. Effects on Biological Parameters

**Figure 3:**
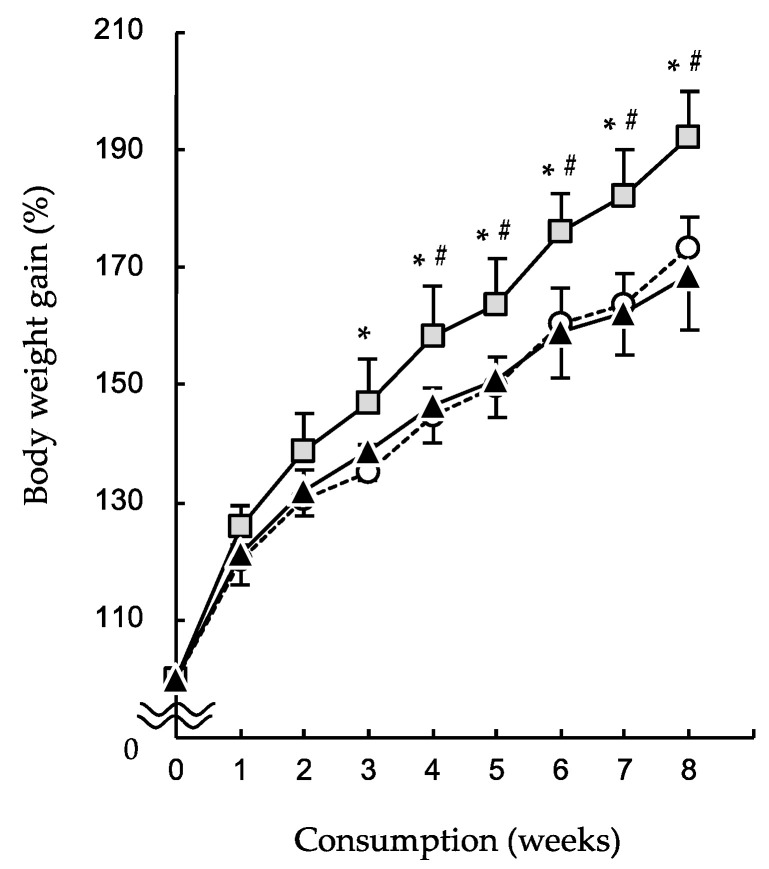
Effects of selectivity for high-fat diets composed of different fat sources on body weight gain. Mice were given two diet boxes: (○) w-SOY; (■) SOY-vs-LARD; and (▲) SOY-vs-FISH. Data are shown as the mean ± SD (*n* = 9). * *p* < 0.01 vs. w-SOY and # *p* < 0.01 vs. SOY-vs-LARD group.

The SOY-vs-LARD group (■) gained significantly more body weight than the w-SOY group (○) from week 3 and the SOY-vs-FISH (▲) group from week 4 (Figure 3). Body weight gains in the w-SOY group and SOY-vs-FISH group increased with similar tendencies. After 8 weeks, the SOY-vs-LARD group (16.1 ± 1.3 g) gained significantly more weight than the w-SOY group (12.8 ± 1.0 g) and the SOY-vs-FISH group (12.0 ± 2.0 g; Table 2). The body mass index showed a similar pattern. The visceral fat was markedly heavier in the SOY-vs-LARD group than in the other two groups, but not significantly. Liver, kidney, and spleen weights were unchanged in all groups.

### 2.4. Effects on Plasma Biochemical and Hepatic Lipid Parameters

Plasma lipid parameters (triglycerides and total cholesterol) were markedly, but not significantly, higher in the following order: SOY-vs-LARD > w-SOY > SOY-vs-FISH groups (Table 3). Plasma total protein levels did not differ among the three groups. Hepatic triglycerides and total cholesterol levels also showed similar tendency with plasma lipid parameters.

### 2.5. Effects on Plasma and Hepatic Fatty Acid Composition

**Figure 4:**
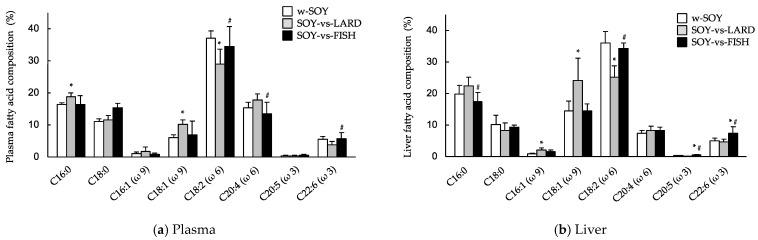
Effects of selectivity of high-fat diets composed of different fat sources on fatty acid compositions in the plasma (**a**) and liver (**b**). w-SOY (□), SOY-vs-LARD (■) and SOY-vs-FISH (■). Data are shown as the mean ± SD (*n* = 9). * *p* < 0.01 vs. w-SOY group; # *p* < 0.01 vs. SOY-vs-LARD group.

In the plasma and liver, the 16:0 (palmitic), 16:1 (palmitoleic), 18:1 (oleic), and 20:4 (arachidonic) fatty acid levels increased, while the 18:2 (linoleic) acid level decreased in the SOY-vs-LARD group (Figure 4). Hepatic 20:5 (eicosapentaenoic acid; EPA) and 22:6 (docosahexaenoic acid; DHA) compositions were significantly higher in the SOY-vs-FISH group than in the other two groups. The plasma EPA and DHA compositions were significantly higher in the SOY-vs-FISH group than in the SOY-vs-LARD group but were similar to those in the w-SOY group.

## 3. Discussion

Mice regulate their food consumption primarily to meet their energy needs, but this system can be overridden by hedonic factors linked to fat consumption [6]. Therefore, daily energy intake under the *ad libitum* feeding condition was expected to be the same among the three groups (Figure 1b). However, mice exhibited selectivity for high-fat diets composed of different fat sources, showing greater selectivity towards the LARD diet than the SOY diet and greater selectivity towards the SOY diet than the FISH diet (Figure 1a). The evidential mechanisms regarding the selectivity for different fat sources remain unclear, but one plausible mechanism is considered: rodents exhibit spontaneous attraction for lipids [12]. The fatty acid transporter, CD36, may regulate this function because it is a membrane receptor that facilitates long-chain fatty acids and is found in rodents’ lingual papillae [14]. Guo et al. reported that CD36 might selectively transport linoleic, arachidonic, and oleic acids, but not palmitic or stearic acids [15]. The major fatty acid compositions of the soybean oil, lard, and fish oil used in this study were linoleic acid (57.15%), oleic acid (40.75%), and palmitic acid (22.3%)/docosahexaenoic acid (28.08%), respectively (Table 1). Different fatty acid compositions may explain why the mice preferentially selected different high-fat diets composed of different fat sources.

Selection of the different diets induced different body weight gains after 8 weeks of consumption. The SOY-vs-LARD group was significantly overweight compared with the w-SOY and SOY-vs-FISH groups (Figure 3 and Table 2). Additionally, an excess body mass index was observed in the SOY-vs-LARD group (Table 2). Triglycerides and total cholesterol levels increased markedly in the plasma and livers of the SOY-vs-LARD group (Table 3). These increases in weight-related parameters in the SOY-vs-LARD group are likely due to an increased omega-6/omega-3 fatty acid ratio. In the past three decades, intake of omega-6 fatty acids has increased, while that of omega-3 fatty acids has decreased, thus increasing the omega-6/omega-3 ratio from 1:1 to 20:1 [16]. This change parallels a significant increase in the prevalence of excess weight gain and obesity [16]. The w-SOY, SOY-vs-LARD, and SOY-vs-FISH groups consumed daily diets containing omega-6/omega-3 ratios of 10.2, 13.0, and 7.3, respectively (Table 1). The mice selectively consumed the LARD diet (omega-6/omega-3 ratio = 41.71) (Table 1), which consequently may have induced excess weight gain. Additionally, the SOY-vs-FISH group gained less weight than the w-SOY group, although the difference was not significant (Table 2). This difference was explained by the omega-6/omega-3 ratio. Dietary fatty acid composition is recognized to affect the body fat composition. For example, Pavlisova et al. reported that hepatic fatty acid compositions are dramatically different between mice consuming a corn oil-based diet rich in unsaturated fatty acids and a lard-based diet rich in saturated fatty acids [17], indicating that body fat compositions are different among the three groups in this study.

Animal studies and human trials have shown that fish oil has a beneficial effect on obesity. For example, rats fed diets containing 20% fish oil gained significantly less weight than rats fed diets containing 20% lard, independently of feeding behavior [18]. Mice who consumed a diet containing 10.5% fish oil gained less weight than those who consumed 4.2% lard + 6.3% safflower oil after 8 weeks of consumption [19]. Du et al. conducted a meta-analysis of randomized controlled trials and found that fish oil supplementation might help reduce abdominal fat [10]. In the present study, the SOY-vs-FISH group consumed >93% of their fat from the SOY diet (Figure 1a). However, their diurnal consumption behavior showed an interesting pattern. Upon *ad libitum* feeding, food consumption in the light period (inactive phase) was low, then increased just before the onset of the dark period (ZT12). The total food consumption for all groups showed two peaks at the beginning and end of the dark period (Figure 2), which was similar to the results of other studies using mice and rats [20,21]. Here, the SOY-vs-FISH group consumed the FISH diet mainly at the end of the dark period (active period), although <7% of the FISH diet was consumed (Figure 2b). High intake of omega-3 fatty acids resulted in higher plasma and liver EPA and DHA levels [22]. Our data indicated that small but selective consumption of the omega-3 fatty acid-rich FISH diet significantly increased hepatic EPA and DHA levels compared with those of the w-SOY and SOY-vs-LARD groups (Figure 4b). The plasma levels showed similar results (Figure 4a). Consumption of these omega-3 fatty acids has been reported to exert many beneficial effects on human health, e.g., improved anti-inflammatory functions, cognitive functions, and antioxidative effects, as well as reduced lipid accumulation [3,23,24]. We could not obtain the mechanism to explain why the mice consumed the diet containing fish oil at the end of the dark period, but such selective consumption of fish oil might affect excess body weight. In order to obtain more detailed evidence, the effects of oral administration of omega-3 fatty-acid-rich fish oil at the end of the active period should be investigated.

Recently, omega-3 fatty acids have been reported to exert beneficial effects on glucose homeostasis. For example, omega-3 polyunsaturated fatty acid intake modulated postprandial glucose metabolism in obese mice through a glucagon-like peptide-1 (GLP-1)-based pathway [25]. EPA treatment increased blood glucose levels after glucose loading in controlled low-fat-diet-fed mice, but improved glucose tolerance in high-fructose/high-fat-diet-fed mice [26]. These observations imply the possibility that selective consumption of omega-3 polyunsaturated fatty-acid-rich fish oil at the end of the day helps the regulation of glucose metabolism on high-fat-diet-induced obesity, but would have a less marked effect under normal conditions. More detailed studies of the underlying effects of selective consumption of fish oil should be undertaken in the future.

## 4. Materials and Methods

### 4.1. Chemicals

Soybean oil and fish oil were obtained from J-Oil Mills, Inc. (Yokohama, Japan). Lard, vitamin mixture (AIN-93-VX), and mineral mixture (AIN-93G-Mix) were obtained from MP Biomedicals, LLC (Irvine, CA, USA). Cellulose, α-cornstarch, β-cornstarch, and sucrose were purchased from Oriental Yeast Co., Ltd. (Tokyo, Japan). Casein, L-cystine, choline bitartrate, and t-butylhydroquinone were obtained from Wako Pure Chemical Industries, Ltd. (Osaka, Japan). All other reagents were of the highest grade available.

### 4.2. Experimental Diets

We used 10% (*w*/*w*) soybean oil containing the AIN-93G-based formula as a controlled low-fat diet, as previously described (Table 4) [27]. We used three high-fat diets containing 30% (*w*/*w*) fat, composed of soybean oil (SOY), lard (LARD), or fish oil (FISH). Table 1 summarizes the fatty acid compositions of fats used in this study. Approximately 200 g of each diet was packed into an aluminum pouch with an oxygen absorber and stored at −20 °C until used.

### 4.3. Animal Experiments

#### 4.3.1. Institutional Approval of the Study Protocol

The Institutional Animal Care and Use Committee of the University of Miyazaki (No. 2020-011-2) approved all animal procedures. This study was conducted in accordance with the Japanese Law for the Humane Treatment and Management of Animals (Law No. 105, 1973), which defines animal experimentation as the use of animals for scientific purposes with the consideration of the 3Rs.

#### 4.3.2. Animals and Housing Environments

Twenty-seven 4-week-old male C57BL/6JJmaSlc mice were obtained from Japan SLC (Shizuoka, Japan), housed three per cage (W 335 mm × L 225 mm × H 135 mm) with paper bedding (Alpha-dri Certified, EPS Ekishin Co., Tokyo, Japan), and permitted free access to deionized water and a controlled low-fat diet. Each cage also contained one gnawing brick (50 mm × 50 mm × 10 mm, LUFA-ITL GmbH, Kiel, Germany). To accurately measure the quantity of food consumed, each diet was stocked in a bait box (Roden CAFE Type M, Oriental Yeast Co., Ltd., Tokyo, Japan). All cages were kept in an air-conditioned room (23 ± 2 °C with 55 ± 5% humidity) under a 12 h dark/light cycle (light period: 09:00–21:00).

#### 4.3.3. Experimental Design

Group housing is widely recommended for male laboratory mice in order to enable them to behave socially, but aggression in group-housed male mice can lead to serious health issues, including injury and death [28,29]. However, Jirkof et al. reported that CD-1 mice can be successfully group-housed for up to 14 weeks, and groups of three mice might be best for reducing minor levels of aggression [30]. Although routine laboratory procedures—such as transfer to a new cage—can be stressful for laboratory animals, handling can help improve rodent welfare by reducing stress and exposure to novel stimuli [31]. Additional materials, such as woody tubes, can add enrichment for rodents [32]. Furthermore, the hedonic behavior of older mice is more vulnerable to social defeat compared with that of younger mice [33]. Therefore, we used younger (4-week-old) mice and housed them three per cage with sufficient bedding and one gnawing brick. During the 1-week acclimation period, each mouse was handled daily using a towel for 1 min to provide human contact. Consequently, the mice showed no severe aggressive behaviors such as wounding, abnormal body-weight reduction, or killing their cage mates, indicating that group housing was suitable for male C57BL/6 mice.

**Figure 5:**
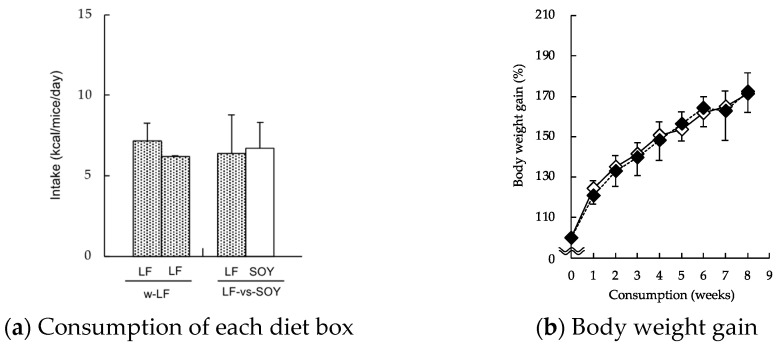
Preliminary experiments for checking the protocol used in this study. After 1 week acclimation, two-diet-box choice test was carried out. Mice were given two different boxes per cage: either two 10% (*w*/*w*) soybean oil-based low-fat (LF) diet boxes (w-LF); or an LF-diet box and a 30% (*w*/*w*) soybean oil-based high-fat diet (SOY) diet box (LF-vs-SOY), and their food consumption (**a**) and body weight gain (**b**) were measured for 8 weeks (◇, w-LF group; ◆, LF-vs-SOY group). Data are shown as the mean ± SD (*n* = 9). No data significantly differed (*p* < 0.01, Tukey–Kramer test), indicating that our protocol is suitable for evaluation of two-diet-box selection.

**Figure 6:**
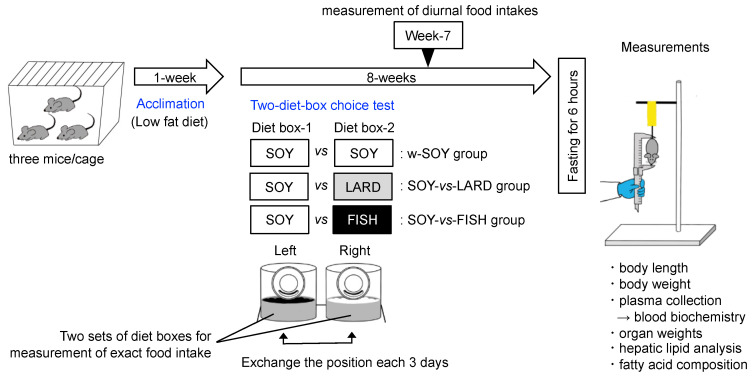
Schematic of the experimental design. After 1 week of acclimation using a 10% soybean oil diet (low-fat diet), two-diet-box choice test was carried out. Mice were given two diet boxes per cage: either two 30% (*w*/*w*) soybean oil-based high-fat (SOY) diet boxes (w-SOY); a SOY-diet box and a 30% (*w*/*w*) lard-based high-fat (LARD) diet box (SOY-vs-LARD); or a SOY-diet box and a 30% (*w*/*w*) fish oil-based high-fat (FISH) diet box (SOY-vs-FISH). Food consumption for each cage, which was average of three mice, was measured 3 times per week during the 8-week experimental period. Each diet box was replaced every 3 days. Additionally, diurnal food intake patterns were analyzed at week 7. After 8 weeks of consumption, body mass index, blood biochemistry, organ weights, hepatic lipid levels, and fatty acid composition were analyzed.

Before starting the main experiments, we checked the appropriateness of our protocol using two diets composed with 10% soybean oil diet (LF) and 30% soybean oil diet (SOY) as shown in Figure 5. These results suggest that mice consumed their diets equally from both diet boxes, and that they did not show selectivity to low-fat or high-fat diets consisting of a single oil (soybean oil). Following this, to evaluate the selection of high-fat diets composed of different fat sources, two different diets in bait boxes were placed in the cages (Figure 6). After 1 week of acclimation using a controlled low-fat diet, the mice were assigned to either the double SOY-box (w-SOY), SOY-box and LARD-box (SOY-vs-LARD), or SOY-box and FISH-box (SOY-vs-FISH) group, with three cages (three mice per cage). Food consumption for each cage, which averaged three mice, was measured 3 times per week during the 8-week experimental period. Diets were refreshed after each measurement, and the body weights of the mice were measured weekly. Additionally, diurnal food intake patterns were analyzed, and the individual diet boxes were weighed every 2 h for 48 h during week 7.

#### 4.3.4. Sample Collection

After *ad libitum* feeding from two diet boxes for 8 weeks, the mice were fasted for 6 h, then anesthetized with isoflurane (1.5%), and their body lengths (from tail root to nose) were measured according to the modified method reported by Novelli et al. [34] during Zeitgeber time (ZT) 4 to ZT6. Here, ZT0 represents the time when the light was turned on at the start of the light period. Hence, the light period lasted from ZT0 to ZT12, and the dark period lasted from ZT12 to ZT24. The body weight just before fasting and body length were used to determine the body mass index using the following Formula (1):

Body mass index = body weight (g)/length (cm^2^) (1)

After measuring the body length, blood was drawn from the abdominal vein and collected in EDTA-coated tubes (Microtainer Tube with EDTA and Microgard Closure, BD, Mississauga, ON, Canada), then centrifuged at 2000× *g* for 90 s at 15 °C. The plasma fraction was stored at −80 °C until further analysis. The liver, kidney, spleen, and visceral fat (epididymal fat + perirenal fat) were weighed. One liver section was flash-frozen in liquid nitrogen and stored at −80 °C for later lipid analysis.

### 4.4. Biochemical Parameters

#### 4.4.1. Blood Biochemistry

Plasma biochemical parameters (triglycerides, total cholesterol, and total protein) were analyzed using a Dri-Chem 4000v chemistry analyzer (Fujifilm Co., Tokyo, Japan) and an individual cartridge slide.

#### 4.4.2. Hepatic Lipid Analysis

Hepatic lipid levels were determined as described previously [35]. Briefly, 200 mg liver samples were homogenized with 1 mL of 50 mM sodium acetate. Subsequently, 6 mL of chloroform-methanol (2:1 [vol/vol]) was added, and the mixture was incubated at 40 °C for 30 min. A 500-μL aliquot of the organic phase was dried using a centrifugal concentrator (CC-105: Tomy Seiko Co., Ltd., Tokyo, Japan). The residues were dissolved in 80 µL of 10% Triton X-100 containing isopropyl alcohol. Triglycerides, total cholesterol, and phospholipid levels were analyzed using individual test kits purchased from Wako Pure Chemical industries, Ltd. (Osaka, Japan)

#### 4.4.3. Plasma and Hepatic Fatty Acid Composition

Plasma fatty acid compositions were analyzed using the modified method reported by Monguchi et al. [36]. Briefly, total fatty acid extraction from 25 µL of plasma and methyl-ester derivatization were performed using the Fatty Acid Methylation kit (Nacalai Tesque, Inc., Kyoto, Japan) per the manufacturer’s instructions. Methyl-ester-derivatized fatty acids were reconstituted with 200 µL of hexane for subsequent analysis.

To analyze the hepatic fatty acid composition, liver samples (200 mg) were homogenized with 1 mL of 50 mM sodium acetate, then extracted using the chloroform-methanol mixture described above. Subsequently, aliquots of the extracts (20 mg liver equivalent) were methyl-ester-derivatized and reconstituted with 200 µL of hexane.

Fatty acids were analyzed using a gas chromatography–flame ionization detector (GC-2010, Shimadzu Co., Kyoto, Japan). The SUPELCOWAXTM 10 capillary column (30 m length × 0.32 mm inner diameter × 0.25-μm film thickness; Sigma-Aldrich, St. Louis, MO, USA) was used to separate the fatty acids. The column oven temperature was elevated from 170 °C to 225 °C, and the separated fatty acid methyl ester was detected using the flame ionization detector. The standard mixture of methyl ester fatty acids (Supelco 37 Component FAME Mix) was obtained from Sigma-Aldrich (St. Louis, MO, USA).

### 4.5. Statistical Analysis

Data are presented as the mean ± standard deviation (SD). Statistical analyses were conducted using Pharmaco ANOVA (ver. 1) and Pharmaco Basic (ver. 16) (Scientist Press Co., Ltd., Tokyo, Japan). Statistical significance among groups was determined using one-way analysis of variance, followed by the Tukey–Kramer post hoc test. For within-group comparisons, the alpha value was set at 0.01.

## 5. Conclusions

In the present study, we investigated the effects of selecting 30% fat diets composed of SOY, LARD, and FISH on metabolic parameters in mice using a self-selection regimen from two diet boxes. The results obtained indicate that they preferred the LARD diet over the SOY diet and the SOY diet over the FISH diet, although the total energy intake was the same for all three groups. Body weight in the SOY-vs-LARD group was significantly higher than that in the w-SOY and SOY-vs-FISH. Additionally, minimal but selective consumption of an omega-3 fatty-acid-rich FISH diet at the end of the active period increased the physiological fatty acid compositions of EPA and DHA in the SOY-vs-FISH group; their metabolic parameters were also lower than the SOY-vs-LARD group. The results obtained in this study imply that consuming small amounts of fish oil at the end of the active period may help prevent excess weight, compared at least to lard consumption.

## Figures and Tables

**Table 1:** Daily consumption of individual fatty acid (mg/mice/day).

Fatty Acids	Common Name	w-SOY	SOY-vs-LARD	SOY-vs-FISH
C12:0	lauric acid	<1.0	<1.0	<1.0
C14:0	myristic acid	<1.0	7.1 ± 0.4	2.5 ± 0.2 ^#^
C16:0	palmitic acid	76.2 ± 1.2	148.8 ± 9.8 *	78.6 ± 8.3 ^#^
C18:0	stearic acid	22.4 ± 0.4	71.6 ± 4.5 *	22.6 ± 2.4 ^#^
C16:1 (ω 9)	palmitoleic acid	<1.0	18.4 ± 1.2	6.0 ± 0.4 ^#^
C18:1 (ω 9)	oleic acid	182.6 ± 2.9	272.3 ± 18.6 *	171.0 ± 18.8 ^#^
C18:2 (ω 6)	linoleic acid	432.0 ± 6.9	263.2 ± 26.0 *	388.0 ± 43.3 ^#^
C18:3 (ω 6)	γ-linolenic acid	<1.0	1.2 ± 0.1	<1.0
C20:4 (ω 6)	arachidonic acid	<1.0	<1.0	<1.0
C18:3 (ω 3)	α-linolenic acid	42.1 ± 0.7	20.3 ± 2.4 *	38.0 ± 4.2 ^#^
C20:5 (ω 3)	eicosapentaenoic acid	<1.0	<1.0	3.0 ± 0.2
C22:6 (ω 3)	docosahexaenoic acid	<1.0	<1.0	12.9 ± 0.9
Sum of saturated fatty acids		99.0 ± 1.6	227.9 ± 14.7 *	103.7 ± 10.9 ^#^
Sum of monounsaturated fatty acids		182.7 ± 2.9	290.7 ± 19.6 *	177.0 ± 19.2 ^#^
Sum of omega-6 fatty acids		432.0 ± 6.9	265.0 ± 26.1 *	389.2 ± 43.4 ^#^
Sum of omega-3 fatty acids		42.2 ± 0.7	20.4 ± 2.4 *	53.9 ± 5.3 ^#^
omega-6/omega-3 ratio		10.2	13.0	7.3

SOY, 30% (*w*/*w*) soybean oil high-fat diet; LARD, 30% (*w*/*w*) lard high-fat diet; FISH, 30% (*w*/*w*) fish oil high-fat diet. Data indicate the mean ± SD (*n* = 3). * *p* < 0.01 vs. w-SOY group; ^#^ *p* < 0.01 vs. SOY-vs-LARD group.

**Table 2:** Effects of self-selection of different high-fat diets on growth and organ weights.

	w-SOY	SOY-vs-LARD	SOY-vs-FISH
Body weight (g)			
week 0	17.5 ± 0.8	17.5 ± 0.6	17.5 ± 1.4
week 8	30.3 ± 1.6	33.7 ± 1.6	29.8 ± 3.2 ^#^
Body weight gain (g)	12.8 ± 1.0	16.1 ± 1.3 *	12.0 ± 2.0 ^#^
Body length (cm)	7.9 ± 0.3	8.1 ± 0.2	8.0 ± 0.1
Body mass index (g/cm^2^)	0.49 ± 0.03	0.52 ± 0.02	0.46 ± 0.03 ^#^
Relative organ weights (g/100 g body weight)			
liver	3.54 ± 0.34	3.31 ± 0.48	3.65 ± 0.64
kidney	1.20 ± 0.16	1.14 ± 0.12	1.19 ± 0.15
spleen	0.31 ± 0.05	0.30 ± 0.07	0.34 ± 0.12
visceral fat	4.59 ± 1.31	5.59 ± 0.49	3.93 ± 1.43

SOY, 30% (*w*/*w*) soybean oil high-fat diet; LARD, 30% (*w*/*w*) lard high-fat diet; FISH, 30% (*w*/*w*) fish oil high-fat diet. Data indicate the mean ± SD (*n* = 9). * *p* < 0.01 vs. w-SOY group; ^#^ *p* < 0.01 vs. SOY-vs-LARD group.

**Table 3:** Effects of self-selection of different high-fat diets on plasma biochemistry parameters and hepatic lipids of individual mice.

	w-SOY	SOY-vs-LARD	SOY-vs-FISH
Plasma biochemistry parameters			
triglycerides (mg/dL)	57.4 ± 14.4	74.3 ± 21.4	48.7 ± 24.0
total cholesterol (mg/dL)	96.5 ± 21.9	109.9 ± 32.4	85.7 ± 33.8
total protein (g/dL)	4.7 ± 0.3	4.7 ± 0.4	4.6 ± 0.5
Hepatic lipids (mg/g liver)			
triglycerides	9.8 ± 1.6	12.5 ± 4.8	8.8 ± 2.1
total cholesterol	3.7 ± 0.7	5.4 ± 2.6	3.9 ± 0.7
phospholipids	5.4 ± 0.9	5.5 ± 0.9	5.0 ± 0.9

SOY, 30% (*w*/*w*) soybean oil high-fat diet; LARD, 30% (*w*/*w*) lard high-fat diet; FISH, 30% (*w*/*w*) fish oil high-fat diet. Data indicate the mean ± SD (*n* = 9). No data significantly differed (*p* < 0.01, Tukey–Kramer test).

**Table 4:** Compositions of the AIN-93G-based experimental diets (%).

	10% Soybean Oil	30% Fat Diet		
		Soybean Oil (SOY)	Lard (LARD)	Fish Oil (FISH)
β-Cornstarch	36.75	16.75	16.75	16.75
α-Cornstarch	13.2	13.2	13.2	13.2
Casein	20.0	20.0	20.0	20.0
Soybean oil	10.0	30.0	-	-
Lard	-	-	30.0	-
Fish oil	-	-	-	30.0
Sucrose	10.0	10.0	10.0	10.0
Cellulose	5.0	5.0	5.0	5.0
Vitamin mixture	1.0	1.0	1.0	1.0
Mineral mixture	3.5	3.5	3.5	3.5
*L*-Cysteine	0.30	0.30	0.30	0.30
Choline bitartrate	0.25	0.25	0.25	0.25
*t*-Butylhydroquinone	0.0014	0.0014	0.0014	0.0014
Energy (kcal/g)	4.098	5.098	5.098	5.098

## Data Availability

No new data were created or analyzed in this study. Data sharing is not applicable to this article.

## Sample Availability

Samples of the compounds are available from the authors.

## References

[1] Elagizi A., Kachur S., Lavie C.J., Carbone S., Pandey A., Ortega F.B., Milani R.V. (2018). An overview and update on obesity and the obesity paradox in cardiovascular diseases. Prog. Cardiovasc. Dis..

[2] Vecchie A., Dallegri F., Carbone F., Bonaventura A., Liberale L., Portincasa P., Fruhbeck G., Montecucco F. (2018). Obesity phenotypes and their paradoxical association with cardiovascular diseases. Eur. J. Intern. Med..

[3] Wang Y.C., McPherson K., Marsh T., Gortmaker S.L., Brown M. (2011). Health and economic burden of the projected obesity trends in the USA and the UK. Lancet.

[4] Nani A., Murtaza B., Sayed Khan A., Khan N.A., Hichami A. (2021). Antioxidant and anti-inflammatory potential of polyphenols contained in mediterranean diet in obesity: Molecular mechanisms. Molecules.

[5] Li H., Zhu Y., Zhao F., Song S., Li Y., Xu X., Zhou G., Li C. (2017). Fish oil, lard and soybean oil differentially shape gut microbiota of middle-aged rats. Sci. Rep..

[6] Hu S., Wang L., Yang D., Li L., Togo J., Wu Y., Liu Q., Li B., Li M., Wang G. (2018). Dietary fat, but not protein or carbohydrate, regulates energy intake and causes adiposity in mice. Cell Metab..

[7] Bray G.A., Lovejoy J.C., Smith S.R., DeLany J.P., Lefevre M., Hwang D., Ryan D.H., York D.A. (2002). The influence of different fats and fatty acids on obesity, insulin resistance and inflammation. J. Nutr..

[8] Yang Y., Du L., Hosokawa M., Miyashita K. (2020). Effect of Spirulina lipids on high-fat and high-sucrose diet induced obesity and hepatic lipid accumulation in C57BL/6J mice. J. Funct. Food.

[9] Na B.R., Lee J.H. (2020). In vitro and in vivo digestibility of soybean, fish, and microalgal oils, and their influences on fatty acid distribution in tissue lipid of mice. Molecules.

[10] Du S., Jin J., Fang W., Su Q. (2015). Does fish oil have an anti-obesity effect in overweight/obese adults? A meta-analysis of randomized controlled trials. PLoS ONE.

[11] Imaizumi M., Takeda M., Suzuki A., Sawano S., Fushiki T. (2001). Preference for high-fat food in mice: Fried potatoes compared with boiled potatoes. Appetite.

[12] Takeda M., Imaizumi M., Fushiki T. (2000). Preference for vegetable oils in the two-bottle choice test in mice. Life Sci..

[13] Mizunoya W., Ohnuki K., Baba K., Miyahara H., Shimizu N., Tabata K., Kino T., Sato Y., Tatsumi R., Ikeuchi Y. (2013). Effect of dietary fat type on anxiety-like and depression-like behavior in mice. Springerplus.

[14] Laugerette F., Passilly-Degrace P., Patris B., Niot I., Febbraio M., Montmayeur J.P., Besnard P. (2005). CD36 involvement in orosensory detection of dietary lipids, spontaneous fat preference, and digestive secretions. J. Clin. Investig..

[15] Guo J., Shu G., Zhou L., Zhu X., Liao W., Wang S., Yang J., Zhou G., Xi Q., Gao P. (2013). Selective transport of long-chain fatty acids by FAT/CD36 in skeletal muscle of broilers. Animal.

[16] Simopoulos A.P. (2016). An increase in the omega-6/omega-3 fatty acid ratio increases the risk for obesity. Nutrients.

[17] Pavlisova J., Bardova K., Stankova B., Tvrzicka E., Kopecky J., Rossmeisl M. (2016). Corn oil versus lard: Metabolic effects of omega-3 fatty acids in mice fed obesogenic diets with different fatty acid composition. Biochimie.

[18] Gondim P.N., Rosa P.V., Okamura D., Silva V.O., Andrade E.F., Biihrer D.A., Pereira L.J. (2018). Benefits of fish oil consumption over other sources of lipids on metabolic parameters in obese rats. Nutrients.

[19] Arai T., Kim H.J., Chiba H., Matsumoto A. (2009). Anti-obesity effect of fish oil and fish oil-fenofibrate combination in female KK mice. J. Atheroscler Thromb.

[20] Chen X., Wang L., Loh D.H., Colwell C.S., Tache Y., Reue K., Arnold A.P. (2015). Sex differences in diurnal rhythms of food intake in mice caused by gonadal hormones and complement of sex chromosomes. Horm. Behav..

[21] Yokoyama D., Tanaka W., Hashizume Y., Tandia M., Sakono M., Shimoi K., Sakakibara H. (2018). Daily consumption of monoglucosyl-rutin prevents high-fat diet-induced obesity by suppressing gastric inhibitory polypeptide secretion in mice. Funct. Food Health Dis..

[22] Balogun K.A., Albert C.J., Ford D.A., Brown R.J., Cheema S.K. (2013). Dietary omega-3 polyunsaturated fatty acids alter the fatty acid composition of hepatic and plasma bioactive lipids in C57BL/6 mice: A lipidomic approach. PLoS ONE.

[23] Liu J.H., Wang Q., You Q.L., Li Z.L., Hu N.Y., Wang Y., Jin Z.L., Li S.J., Li X.W., Yang J.M. (2020). Acute EPA-induced learning and memory impairment in mice is prevented by DHA. Nat. Commun..

[24] Soni N., Ross A.B., Scheers N., Nookaew I., Gabrielsson B.G., Sandberg A.S. (2019). The omega-3 fatty acids EPA and DHA, as a part of a murine high-fat diet, reduced lipid accumulation in brown and white adipose tissues. Int. J. Mol. Sci..

[25] Pavlisova J., Horakova O., Kalendova V., Buresova J., Bardova K., Holendova B., Plecita-Hlavata L., Vackova S., Windrichova J., Topolcan O. (2020). Chronic n-3 fatty acid intake enhances insulin response to oral glucose and elevates GLP-1 in high-fat diet-fed obese mice. Food Funct..

[26] El-Fayoumi S.H., Mahmoud A.A.A., Fahmy A., Ibrahim I. (2020). Effect of omega-3 fatty acids on glucose homeostasis: Role of free fatty acid receptor 1. Naunyn-Schmiedeberg’s Arch. Pharmacol..

[27] Matsuyama H., Tanaka W., Yokoyama D., Matsumoto S., Sano T., Yamashita T., Nishimura S., Sakono M., Sakakibara H. (2020). Suitability of a 10% fat diet for use in time-restricted feeding experiments with C57BL/6 mice. Bioact. Compd. Health Dis..

[28] Kappel S., Hawkins P., Mendl M.T. (2017). To group ornot to group? Good practice for housing male laboratory mice. Animals.

[29] Mertens S., Vogt M.A., Gass P., Palme R., Hiebl B., Chourbaji S. (2019). Effect of three different forms of handling on the variation of aggression-associated parameters in individually and group-housed male C57BL/6NCrl mice. PLoS ONE.

[30] Jirkof P., Bratcher N., Medina L., Strasburg D., Ebert P., Gaskill B.N. (2020). The effect of group size, age and handling frequency on inter-male aggression in CD 1 mice. Sci. Rep..

[31] Swennes A.G., Alworth L.C., Harvey S.B., Jones C.A., King C.S., Crowell-Davis S.L. (2011). Human handling promotes compliant behavior in adult laboratory rabbits. J. Am. Assoc. Lab. Anim. Sci..

[32] Eskola S., Lauhikari M., Voipio H.M., Nevalainen T. (1999). The use of aspen blocks and tubes to enrich the cage environment of laboratory rats. Scand. J. Lab. Anim. Sci..

[33] Oizumi H., Kuriyama N., Imamura S., Tabuchi M., Omiya Y., Mizoguchi K., Kobayashi H. (2019). Influence of aging on the behavioral phenotypes of C57BL/6J mice after social defeat. PLoS ONE.

[34] Novelli E.L., Diniz Y.S., Galhardi C.M., Ebaid G.M., Rodrigues H.G., Mani F., Fernandes A.A., Cicogna A.C., Novelli Filho J.L. (2007). Anthropometrical parameters and markers of obesity in rats. Lab. Anim..

[35] Takashima M., Tanaka W., Matsuyama H., Tajiri H., Sakakibara H. (2021). Maternal quercetin consumption during pregnancy may help regulate total cholesterol/HDL-cholesterol ratio without effect on cholesterol levels in male progeny consuming high-fat diet. Nutrients.

[36] Monguchi T., Hara T., Hasokawa M., Nakajima H., Mori K., Toh R., Irino Y., Ishida T., Hirata K.I., Shinohara M. (2017). Excessive intake of trans fatty acid accelerates atherosclerosis through promoting inflammation and oxidative stress in a mouse model of hyperlipidemia. J. Cardiol..

